# Preserving Fertility in Young Women: Triple‐Step Strategy for Managing Giant Endometriomas With Minimal AMH Decline—A Case Report

**DOI:** 10.1155/crog/9079350

**Published:** 2026-04-28

**Authors:** Arry Soryadharma, Hanom Husni Syam, Mulyanusa Amarullah Ritonga

**Affiliations:** ^1^ Reproductive Endocrinology and Infertility Division, Department of Obstetrics and Gynecology, Faculty of Medicine, Universitas Padjadjaran–Dr. Hasan Sadikin General Hospital, Bandung, West Java Province, Indonesia, unpad.ac.id

**Keywords:** AMH, case report, endometrioma, infertility, ovarian reserve, triple-step procedure

## Abstract

Endometriomas are a frequent cause of infertility in reproductive‐aged women, often requiring surgery. However, cystectomy for large endometriomas may reduce ovarian reserve, as indicated by decreased anti‐Müllerian hormone (AMH) levels. This case report presents a triple‐step approach to managing a 20‐year‐old woman with bilateral endometriomas and infertility, focusing on preserving ovarian function. The patient, with primary infertility for 2 years and large bilateral endometriomas (10 cm on the right ovary and 5 cm on the left), underwent a staged treatment. Step 1 involved laparoscopic drainage of the cysts, followed by 3 months of GnRH agonist therapy to shrink the cysts and suppress endometriosis. Step 2 was a laparoscopic cystectomy performed carefully to minimize ovarian tissue loss. Step 3 included 3 more months of postoperative GnRH agonist therapy to suppress residual disease and aid ovarian recovery. Predrainage AMH was 3.27 ng/mL, decreased slightly to 3.17 ng/mL at 6 months after surgery, and was 2.54 ng/mL at 12 months of follow‐up, indicating minimal ovarian reserve depletion. The patient resumed regular menstrual cycles 3 months postoperatively and conceived within 1 year, with no evidence of endometrioma recurrence observed. This case highlights the effectiveness of the triple‐step approach in managing giant endometriomas while preserving fertility. Combining medical and surgical methods reduced AMH depletion and improved reproductive outcomes. This strategy may be a valuable option for young women with endometriomas, though further research is needed to confirm its long‐term benefits.

## 1. Introduction

Large endometrioma, with a diameter of > 5 cm, is an estrogen‐dependent disorder marked by the ectopic implantation of functioning uterine tissue (endometrial glands and stroma) outside of the uterine cavity, which results in the formation of a cyst. This forms when endometrial tissue, which is the mucous membrane lining the inner layer of the uterine wall, grows in the ovaries. [1] This tissue, which usually lines the inner surface of the uterus and sheds during menstruation, can implant and develop in numerous pelvic and abdominal sites, causing a variety of symptoms and difficulties. Endometriomas can range in size from 0.75 to 8 in. and are usually packed with dark, reddish‐brown blood. Because of the thick, dark brown fluid within, they are frequently called “chocolate cysts.” Endometriomas, a frequent ovarian manifestation of endometriosis, impact between 17%–44% of women with endometriosis diagnoses. Another study reported that large endometrioma affects millions of women globally, with estimates indicating that up to 10% of reproductive‐age women are afflicted. [2] According to a previous study, 260 out of the 1191 women with subfertility had endometriotic lesions, making the incidence of endometriosis among these women 21.8%. Furthermore, women between the ages of 15 and 50 had an incidence of 0.14% for endometriosis. A thorough study conducted over 15 years revealed that the yearly incidence of pelvic endometriosis in women of reproductive age ranges from 0.1% to 0.15%. [3, 4]

Conventional management of large endometriomas commonly involves single‐stage laparoscopic cystectomy; however, this approach may compromise ovarian reserve due to inadvertent removal of healthy ovarian tissue. To mitigate this risk, staged and three‐step strategies combining cyst drainage, hormonal suppression, and delayed definitive surgery have been proposed. Donnez et al. described a three‐step approach consisting of laparoscopic cyst drainage with biopsy confirmation, followed by 12 weeks of gonadotropin‐releasing hormone (GnRH) agonist therapy and subsequent cyst wall ablation using a CO_2_ laser. [5] In contrast, our approach replaces ablation with laparoscopic cystectomy in the final stage, with particular emphasis on meticulous surgical technique to preserve ovarian tissue, especially in cases of large bilateral endometriomas. In this report, we present a modified triple‐step strategy involving initial cyst aspiration, GnRH agonist‐induced downregulation, and interval reassessment prior to definitive cystectomy, aimed at optimizing surgical conditions while minimizing ovarian reserve depletion.

The triple‐step method, a procedure carried out at Hasan Sadikin Hospital Bandung, consists of laparoscopic aspiration of cyst fluid, three cycles of GnRH agonist (leuprolide acetate 3.75 mg) administration, and subsequent laparoscopic cystectomy. After the last GnRH agonist administration, a re‐evaluation was carried out regarding the presence of endometriosis cysts and antral follicle count (AFC) levels. This minimally invasive technique is used to identify and treat endometriotic cysts, also known as endometriomas or ovarian chocolate cysts. Aspiration of cyst fluid using minimally invasive laparoscopic surgery may be performed for diagnostic purposes or for the management of symptoms such as pelvic pain and dysmenorrhea. [6] GnRH agonists, a class of medications commonly used in the management of large endometriomas, act on the pituitary gland to suppress the secretion of gonadotropins (luteinizing hormone and follicle‐stimulating hormone). This leads to downregulation of ovarian estrogen production and induction of a reversible state of hypoestrogenism. [7] Laparoscopic cystectomy after downregulation may be performed for cysts < 3 cm in size, thereby minimizing damage to healthy ovarian tissue. This procedure involves removal of endometriotic cysts while preserving normal ovarian tissue to alleviate symptoms, improve fertility outcomes, and reduce the risk of disease recurrence. [8]

The patient′s reproductive potential and oocyte function, both qualitatively and quantitatively, are reflected in the ovarian reserve. Endometriomas can impact ovarian reserve in two ways: either by compressing the cyst and reducing circulation in the ovarian cortex, which results in follicle loss, or by creating an inflammatory environment within the cyst walls, which damages follicles. The most popular and trustworthy quantitative ovarian reserve measures are serum anti‐Müllerian hormone (AMH) levels and AFC. To assess ovarian reserve, AMH levels are interpreted with the patient′s age and other clinical factors. Generally, higher AMH levels indicate a more significant ovarian reserve, whereas lower levels may indicate diminished ovarian reserve. [9] However, it is essential to consider that AMH levels decline, and interpretation should be age‐specific. [10] The AFC is interpreted with the patient′s age and other clinical factors to assess ovarian reserve. Generally, a higher AFC indicates a more significant ovarian reserve, whereas a lower AFC may suggest diminished ovarian reserve. However, it is essential to consider that AFC declines with age, reflecting the natural depletion of ovarian follicles over time. The AFC examination is performed before aspiration and 3 months after cystectomy because recovery typically occurs within 3–6 months. [11]

Based on those explanations, this case report describes the use of a triple‐step approach to manage a young woman with bilateral endometriomas and infertility, aimed at preserving ovarian reserve and improving fertility outcomes.

## 2. Case Report

This study reports a case of a 20‐year‐old married woman with severe dysmenorrhea, a 2‐year history of primary infertility, and large bilateral endometriomas (10 cm in the right ovary and 5 cm in the left ovary). As part of the infertility evaluation, the partner′s semen analysis showed normal macroscopic and microscopic findings, and bilateral tubal patency was confirmed intraoperatively. This patient underwent a triple‐step procedure. The first step involved laparoscopic drainage of the endometriomas, followed by 3 months of GnRH agonist therapy to reduce cyst size and suppress endometriosis. The second step was laparoscopic cystectomy, performed with meticulous techniques to minimize ovarian tissue loss. The third step included postoperative GnRH agonist therapy for 3 months to suppress residual disease and promote ovarian recovery.

**Figure 1:**
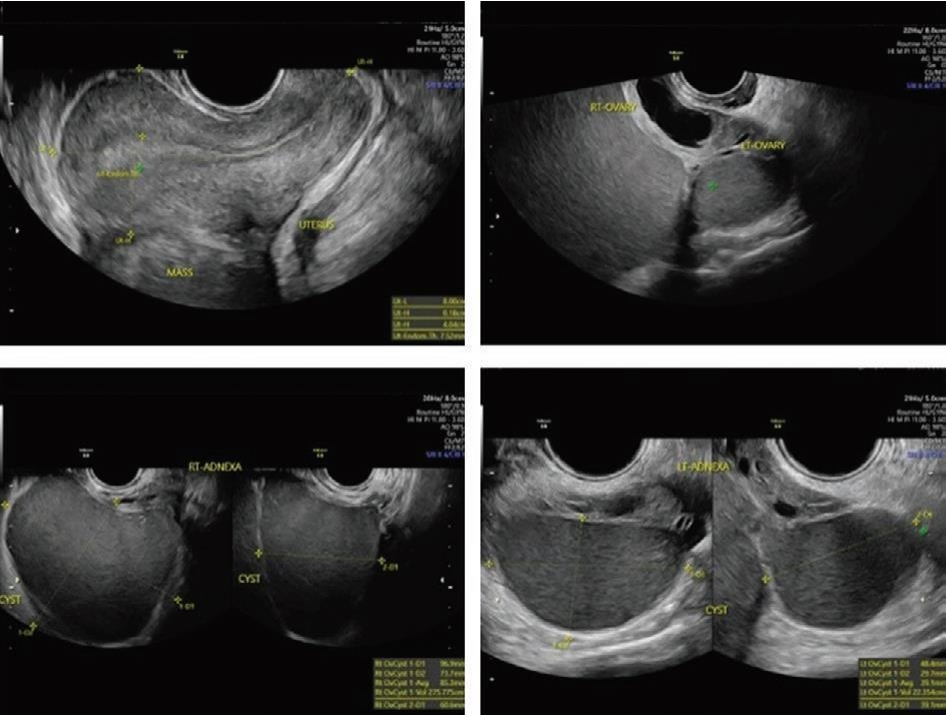
Pelvic ultrasound examination prior to laparoscopic drainage demonstrating bilateral ovarian endometriomas. A large right ovarian endometrioma measuring approximately 10 cm occupied most of the ovary, precluding visualization of antral follicles, whereas the left ovary showed a 5‐cm endometrioma with preserved AFC. Associated pelvic adhesions were also noted.

Figure 1 shows the patient′s pelvic ultrasound findings prior to laparoscopic drainage. The examination revealed bilateral ovarian endometriomas with associated pelvic adhesions. The right ovary was occupied by a 10‐cm endometrioma, precluding visualization of antral follicles, whereas the left ovary contained a 5‐cm endometrioma with a preserved AFC. The patient′s predrainage AMH level was 3.27 ng/mL. Following multidisciplinary discussion and debate during a local fertility conference, a triple‐step surgical approach was selected to relieve symptoms while optimizing preservation of ovarian reserve.

**Figure 2:**
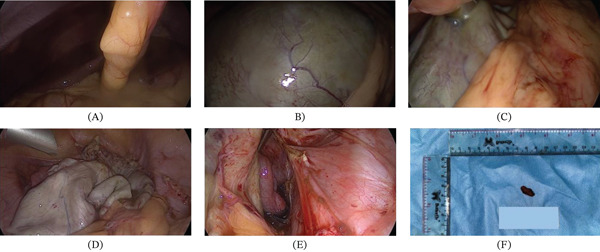
Intraoperative laparoscopic findings during the first step of the triple‐step procedure. (A) Laparoscopic view of the liver surface. (B) Large right ovarian, measuring 10 cm, endometrioma prior to drainage. (C) Direct trocar drainage of the large right ovarian endometrioma. (D) Operative field after drainage of the large right ovarian endometrioma, showing decompression of the cyst. (E) Intraoperative right tubal patency test demonstrating tubal patency. (F) Gross specimen of endometrioma cyst wall obtained intraoperatively and submitted for histopathological examination.

The laparoscopic findings are presented in Figure 2, accurate findings compared with ultrasound are there: a 10‐cm endometrioma on the right and 5 cm on the left ovary. Cysts aspiration was performed followed by adhesiolysis of the bilateral ovarian suspensory ligaments and ovarian fossa; we did wash the pelvic and abdominal cavity thoroughly, and before the operation ended, we performed biopsy of the endometrioma and gel barrier was given on the potential site of readhesion.

**Figure 3:**
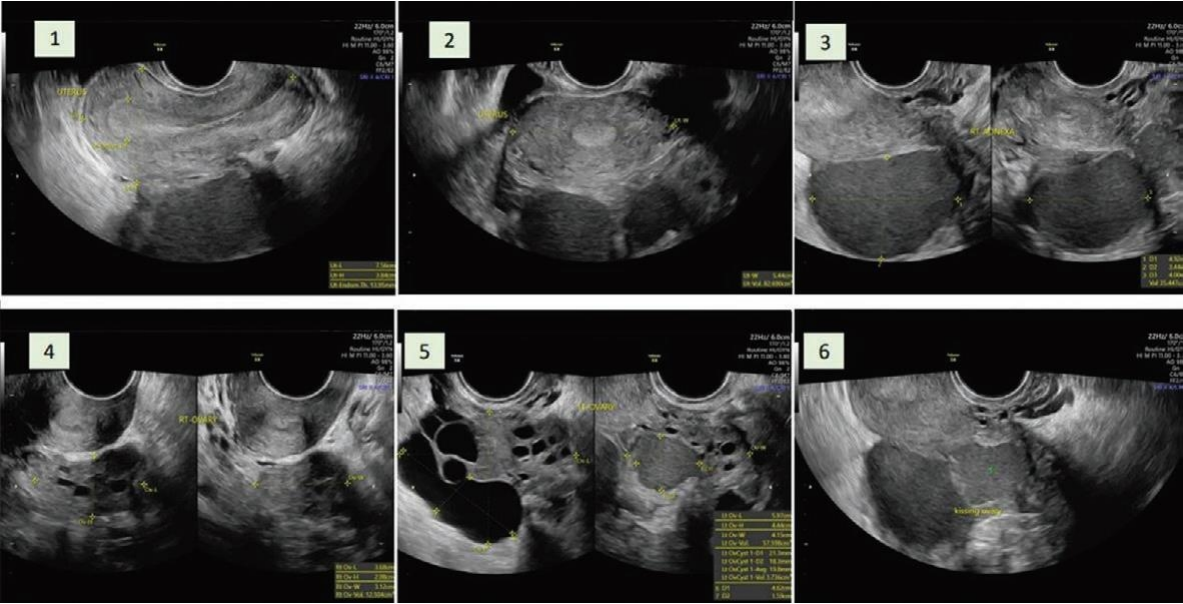
Pelvic ultrasound performed after three cycles of gonadotropin‐releasing hormone (GnRH) agonist therapy, demonstrating significant reduction in cyst size to approximately 2 cm in the right ovary and 4 cm in the left ovary. Following cyst reduction, antral follicle count could be assessed, revealing six antral follicles in the right ovary and nine in the left ovary.

After close monitoring and completion of three cycles of GnRH agonist therapy (leuprolide acetate 3.75 mg), a repeat ultrasound examination was performed prior to cystectomy. This demonstrated a reduction in cyst size to 4 cm in the left ovary and 2 cm in the right ovary (Figure 3). Pelvic adhesions persisted but were not associated with new or worsening symptoms. Although AMH levels were not reassessed at this stage, AFC could be reliably evaluated following cyst reduction, revealing six antral follicles in the right ovary and nine in the left ovary.

**Figure 4:**
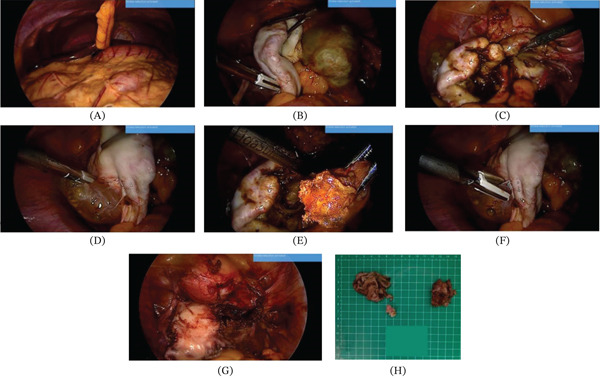
Bilateral laparoscopic cystectomy with adhesiolysis and deep endometriosis resection. (A) Liver and teres hepatic ligament. (B) Residual endometrioma with distorted tubo‐ovarian anatomy. (C) Right cyst wall dissection (right cystectomy). (D) Adhesiolysis and ovarian mobilization. (E) Left cystectomy with removal of endometriotic tissue. (F) Continued adhesiolysis and ovarian mobilization. (G) Final operative field after bilateral cystectomy and adhesiolysis. (H) Excised cyst wall specimens.

Intraoperative findings during second‐look laparoscopy were consistent with the ultrasound results, including ovarian fossa readhesions, thickened cyst walls, and deep endometriosis involving the bilateral uterosacral ligaments (Figure 4); consequently, bilateral cystectomy, adhesiolysis, and resection of deep endometriosis were performed, followed by thorough irrigation of the pelvic and abdominal cavities.

Following completion of the triple‐step strategy, the patient was monitored longitudinally for ovarian reserve and clinical outcomes. Serum AMH levels decreased modestly from 3.27 ng/mL prior to cyst drainage to 3.17 ng/mL at 6 months after cystectomy and 2.54 ng/mL at 12 months of follow‐up. All AMH measurements were performed in the same laboratory using an electrochemiluminescence immunoassay (ECLIA) method and were obtained irrespective of menstrual cycle phase, given the relatively low intracycle fluctuation of AMH levels. Clinically, the patient reported resolution of dysmenorrhea and achieved spontaneous pregnancy within 1 year following surgery.

## 3. Discussion

This study found a similar age profile to Pacchiarotti et al., with a mean age of 33.6 years. [10] Large endometrioma predominantly affects women of reproductive age (25–40 years), with decreased incidence after menopause due to hormonal changes and cessation of menstruation. [12] Nevertheless, postmenopausal women may still present with persistent endometriotic lesions and related symptoms, particularly when disease originates during reproductive years. [13]

Clinical manifestations vary with age; younger women typically present with pelvic pain, dysmenorrhea, dyspareunia, and infertility, whereas diagnosis in older women may be more challenging due to hormonal changes, reduced cyclic symptoms, and altered pelvic anatomy. In addition, imaging modalities such as transvaginal ultrasound and magnetic resonance imaging may be less accurate in such contexts. Therefore, laparoscopy remains the gold standard for diagnosis, allowing direct visualization and histological confirmation. [14, 15] In this case, the patient presented with typical symptoms of pelvic pain and dysmenorrhea, along with altered pelvic anatomy, while maintaining a relatively preserved ovarian reserve as reflected by AMH levels.

The patient had a 2‐year history of primary infertility, consistent with the established association between endometrioma and impaired fertility, which affects approximately 30%–50% of women with endometriosis. [16, 17] Endometrioma contributes to infertility through multiple mechanisms, including disruption of ovulation, inflammatory changes within the pelvic environment, tubal dysfunction, and distortion of pelvic anatomy. [18–20] These factors may impair oocyte quality, fertilization, embryo transport, and implantation. Management of endometrioma‐associated infertility requires a comprehensive and individualized approach involving medical therapy, surgical intervention, and assisted reproductive technologies (ART) when indicated. [16, 17] Although secondary infertility may arise through similar mechanisms, it is additionally influenced by disease progression and the impact of prior treatments on ovarian function and pelvic anatomy. [21–23] In the present case, the patient′s primary infertility, combined with large bilateral endometriomas, highlights the need for a fertility‐preserving strategy.

The patient presented with large bilateral endometriomas measuring 10 and 5 cm, exceeding the mean cyst diameter reported by Ramachandran (4.34 ± 1.32 cm) [24] The size of endometriomas is clinically significant, as larger cysts are more likely to cause pelvic pain, infertility, and distortion of surrounding structures. In addition, larger cysts may increase surgical complexity and the risk of inadvertent damage to healthy ovarian tissue. Consequently, cyst size plays a crucial role in determining management strategies. Although small, asymptomatic cysts may be managed conservatively, larger or symptomatic endometriomas often require surgical intervention, particularly in patients seeking fertility [24, 25] In this context, careful surgical planning is essential to balance effective disease removal with preservation of ovarian reserve.

Assessment of ovarian reserve using AFC is an important component in the evaluation of patients with endometrioma, particularly those undergoing surgical intervention. However, accurate AFC assessment may be challenging in the presence of large endometriomas due to distortion of ovarian architecture and obscuration of follicles. Ramachandran reported relatively low baseline AFC values with subsequent improvement following cystectomy. [24] Similar observations have been reported by Wahd et al. and in a meta‐analysis by Tian et al., highlighting that AFC may improve following surgical treatment of endometriomas as cyst‐related compression and inflammation are reduced. [11, 26] In the present case, baseline AFC prior to the first procedure was difficult to quantify due to large bilateral cysts. Following laparoscopic drainage and three cycles of GnRH agonist therapy, cyst size reduction allowed improved visualization, with AFC values of six in the right ovary and nine in the left ovary. These findings compare favorably with previously reported posttreatment AFC values and suggest preservation of ovarian follicular reserve. This observation supports the rationale of a staged or triple‐step approach, in which initial cyst reduction enhances subsequent surgical precision and minimizes damage to healthy ovarian tissue. [11, 27, 28]

AMH is widely regarded as a reliable biomarker of ovarian reserve, produced by granulosa cells of preantral and small antral follicles. One advantage of AMH is its relative stability throughout the menstrual cycle, allowing measurement at any time. [9] However, interpretation of longitudinal AMH changes requires careful consideration of both analytical and biological variability. Previous studies have demonstrated a decline in AMH following cystectomy, suggesting a potential impact of surgery on ovarian reserve. [24] Similarly, Romanski et al. described a mean AMH level of 2.9 ± 2.5 ng/mL, underscoring the wide interindividual variability of AMH values among reproductive‐age women. Furthermore, longitudinal data, including those from Depmann et al., indicate that AMH levels may vary by approximately 20%–30% annually in reproductive‐age women, reflecting physiological fluctuation rather than true ovarian reserve depletion. [29, 30] Consequently, small changes in AMH that fall within this expected variability range may not represent clinically meaningful ovarian damage but instead reflect normal variation, emphasizing the importance of considering the minimal detectable change when evaluating serial measurements.

In this case, AMH levels declined modestly from 3.27 ng/mL prior to treatment to 3.17 ng/mL at 6 months and 2.54 ng/mL at 12 months following completion of the triple‐step therapy. The minimal decline observed at 6 months (approximately 3%) is well below the expected range of biological variability and is unlikely to represent clinically significant ovarian damage. Even the 12‐month decline (approximately 22%) remains within the reported range of normal year‐to‐year variation. Importantly, no abrupt or disproportionate decrease in AMH was observed, in contrast to the more pronounced declines reported after conventional single‐stage cystectomy. These findings suggest that the staged surgical and medical approach used in this case may help preserve ovarian reserve. All AMH measurements were performed using an ECLIA on the cobas e 402 platform (Roche Diagnostics); however, the absence of detailed interassay and intra‐assay variability data represents a limitation, as it may affect the precision of longitudinal comparisons.

GnRH agonists, such as leuprorelin acetate, play an important role in the management of moderate to severe endometriosis by inducing pituitary desensitization and suppressing gonadotropin release, resulting in a hypoestrogenic state. [31, 32] This leads to a temporary reduction in estrogen‐dependent symptoms, including pelvic pain and dysmenorrhea, as well as a decrease in cyst size. Treatment duration is generally limited to 3–6 months due to hypoestrogenic adverse effects, particularly bone mineral density loss. [7] Although GnRH agonists are effective for symptom control and preoperative optimization, they do not directly enhance fertility, and symptoms may recur after discontinuation without further intervention. [33] In the present case, preoperative GnRH agonist therapy contributed to cyst size reduction, facilitating subsequent surgical management.

Recurrence remains an important consideration in the management of endometrioma. It may occur due to residual disease, ongoing hormonal stimulation, or disease progression. [34–36] Recurrence can significantly impact quality of life and reproductive outcomes, emphasizing the importance of complete but fertility‐preserving surgical treatment. ART, particularly in vitro fertilization (IVF), represent an effective option for patients with endometrioma‐related infertility, especially when other treatments fail or when significant anatomical distortion is present. [37–39] However, access to ART may be limited by financial or resource constraints. In this case, the patient was unable to pursue IVF or intrauterine insemination due to financial limitations. Therefore, the use of a staged surgical approach aimed at preserving ovarian function was particularly important, and the achievement of spontaneous pregnancy within 1 year further supports its clinical utility.

## 4. Conclusion

This case demonstrates the effectiveness of the triple‐step approach in managing giant endometriomas while preserving ovarian reserve. The integration of medical and surgical interventions minimized the risk of AMH depletion and enhanced fertility outcomes. This strategy offers a promising option for young women with endometriomas and infertility, although further studies are needed to validate its long‐term efficacy and generalizability.

## Funding

This publication charge is funded by the Universitas Padjadjaran through the Indonesian Endowment Fund for Education (LPDP) on behalf of the Indonesian Ministry of Higher Education, Science and Technology and managed under the EQUITY Program (Contract Nos. 4303/B3/DT.03.08/2025 and 3927/UN6.RKT/HK.07.00/2025).

## Disclosure

Each author has indicated that she has met the journal′s requirements for authorship.

## Ethics Statement

Ethics committee approval was not required for this single case report according to the institutional policy of our center.

## Consent

Written informed consent was obtained from the patient for publication of anonymized clinical data and accompanying images, in accordance with institutional and journal requirements.

## Conflicts of Interest

The authors declare no conflicts of interest.

## Data Availability

All data pertaining to this case are included within the manuscript.
